# Statins and Cardiomyocyte Metabolism, Friend or Foe?

**DOI:** 10.3390/jcdd10100417

**Published:** 2023-10-02

**Authors:** Tim Somers, Sailay Siddiqi, Wim J. Morshuis, Frans G. M. Russel, Tom J. J. Schirris

**Affiliations:** 1 Department of Cardiothoracic Surgery, Radboud University Medical Center, 6500 HB Nijmegen, The Netherlands; 2 Division of Pharmacology and Toxicology, Department of Pharmacy, Radboud University Medical Center, 6500 HB Nijmegen, The Netherlands; 3 Radboud Center for Mitochondrial Medicine, Radboud University Medical Center, 6500 HB Nijmegen, The Netherlands

**Keywords:** statins, beneficial and adverse effects, cardiomyocytes, metabolism, clinical translation

## Abstract

Statins inhibit HMG-CoA reductase, the rate-limiting enzyme in cholesterol synthesis, and are the cornerstone of lipid-lowering treatment. They significantly reduce cardiovascular morbidity and mortality. However, musculoskeletal symptoms are observed in 7 to 29 percent of all users. The mechanism underlying these complaints has become increasingly clear, but less is known about the effect on cardiac muscle function. Here we discuss both adverse and beneficial effects of statins on the heart. Statins exert pleiotropic protective effects in the diseased heart that are independent of their cholesterol-lowering activity, including reduction in hypertrophy, fibrosis and infarct size. Adverse effects of statins seem to be associated with altered cardiomyocyte metabolism. In this review we explore the differences in the mechanism of action and potential side effects of statins in cardiac and skeletal muscle and how they present clinically. These insights may contribute to a more personalized treatment strategy.

## 1. Introduction

Cardiovascular diseases remain a leading cause of morbidity and mortality worldwide. High blood cholesterol levels, and especially low-density lipoprotein (LDL) cholesterol, are one of the major contributing factors in the pathophysiological mechanism. Consequently, lowering LDL cholesterol levels has shown to effectively reduce the incidence of cardiovascular disease, in particular, coronary artery disease and cerebral strokes [1,2,3]. This gave rise to the development of a variety of cholesterol-lowering drugs, including niacin, fibrates, ezetimibe, and statins, as well as the more recently introduced cholesteryl ester transfer protein (CEPT) and proprotein convertase subtilisin-kexin (PCSK) 9 inhibitors [4].

**Figure 1:**
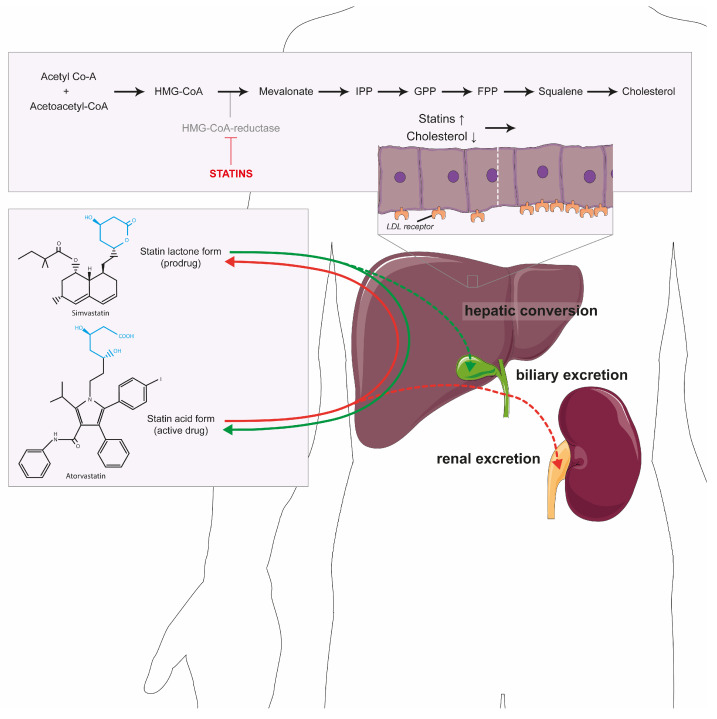
**Statins and the cholesterol pathway.** The conversion of acetyl-CoA by HMG-CoA reductase leading to the production of cholesterol. Statins (in red) inhibit this enzyme, not only leading to less cholesterol, but also isoprenoids, sterols and steroids. The reduction in cholesterol leads to upregulation of the LDL receptor in hepatocytes (displayed in the panel above the liver). Statins are bidirectionally converted in the liver from the prodrug (lactone) into the active form (acid) or back. The acid form is primarily excreted in the urine (dotted red line) and the lactone form into bile (dotted green line). Abbreviations: LDL: low-density lipoprotein; IPP: isopentenyl pyrophosphate; GPP: geranyl diphosphate; FPP: farnesyl diphosphate.

Because of their effectiveness, statins are the most used cholesterol-lowering drugs, which has positioned them as the cornerstone of cardiovascular disease treatment. At the molecular level, their effectivity is based on the inhibition of 3-hydroxy-3-methylglutaryl-coenzyme A (HMG-CoA) reductase, which is the rate-limiting step in the mevalonate pathway (Figure 1, adapted from [5]). Because cholesterol is one of the end products, inhibition of this pathway is a good therapeutic target [5]. This directly reduces blood cholesterol levels, as de novo cholesterol synthesis is one of the major sources of cholesterol besides dietary intake. In addition, low intrahepatic cholesterol concentrations lead to an increased uptake of LDL cholesterol from the central circulation, which further reduces blood cholesterol levels.

Statin effectivity has been demonstrated in a large number of clinical trials, for which a meta-analysis was performed by the Cholesterol Treatment Trialists’ collaboration including 26 randomized trials and over 170,000 patients. Upon statin use, significant reductions were observed in coronary mortality (RR 0.78), myocardial infarction or coronary death (RR 0.76), fatal or non-fatal stroke (RR 0.85) and all these events combined as major vascular events (RR 0.79) [6]. Overall, statins reduced cardiovascular events by 20 percent per mmol/L reduction in LDL cholesterol [6,7]. This reduction also holds for diabetic, hypertensive and non-dyslipidemia patients as well as for individuals with low cardiovascular risk [8,9,10].

In general, statins are well tolerated with a good safety profile, but various adverse effects have been observed, of which muscle complaints are most common [11]. These muscle complaints are experienced by 7 to 29 percent of all 180 million statin users and vary from common muscle stiffness up to rare life-threatening cases of rhabdomyolysis [10,12,13]. Interestingly, these rates are significantly higher than the 1 to 5 percent reported in randomized clinical trials [14,15]. This striking difference is mainly explained by pre-randomization (i.e., excluding patients with statin intolerance), exclusion of muscle complaints from another cause (e.g., physical exercise) in randomized control trials, the absence of an objective measurement for statin-induced muscle complaints, and fundamental differences between both patient groups, including age [16,17]. On the other hand, it is important to realize that a nocebo effect (i.e., information on possible adverse effects induce the experience of these effects) may have a significant contribution to the rates seen in observational studies [15]. A variety of molecular mechanisms have been proposed to explain statin-induced muscle complaints, including disturbed calcium homeostasis, decreased protein prenylation and decreased coenzyme Q_10_ synthesis (CoQ_10_) [18]. Besides effects on CoQ_10_, mitochondrial dysfunction is expected to play a pivotal role. In this respect, we demonstrated that statin lactones inhibit the third complex of the mitochondrial respiratory chain (CIII) [19]. Statin lactones can be formed upon conversion of the pharmacologically active heptanoic acid side chain into a lactone ring by various hepatic UDP-glucuronosyltransferases (UGTs) [20,21]. This lactonization step enhances the biliary excretion of statins as the acid form is cleared renally (Figure 1). Importantly, a high conversion rate leading to high plasma concentrations of the lactone metabolites has been associated with statin-induced muscle complaints [22], which emphasizes the clinical relevance of CIII inhibition by these lactones. The observed CIII inhibition, and associated decreased mitochondrial ATP production, could also be observed in patients with severe statin-induced adverse muscle effects [19,23]. Interestingly, CIII inhibition correlated with the severity of the muscle complaints, which emphasizes the etiologic relevance of this off target.

While the adverse effects of statins on skeletal muscle received much attention, remarkably little is known about the effects on the heart, our body’s most hard-working muscle. Negative effects of statins on the mitochondrial function of cardiomyocytes have, however, been recently described in vitro and in vivo (i.e., in mice) [24], but have so far not been reported clinically. Statins have also been shown to beneficially affect cardiomyocyte function and metabolic activity [25,26,27,28,29]. This raises the question of what could explain these contrasting results between various model systems and within the clinical setting. Here, we discuss all previously reported effects of statins on cardiac muscle using pharmacokinetic, morphological, physiological and metabolic differences between skeletal and myocardial tissue that may explain the differences observed between these tissues. Finally, we will reflect on the consequences of these effects for statin therapy in specific patient groups and the potential need for a more personalized therapy.

## 2. Cholesterol Synthesis and Beyond

Cholesterol synthesis is initiated by the conversion of acetyl CoA and acetoacetyl-CoA by HMG-CoA synthase into HMG-CoA, which is converted into mevalonate by HMG-CoA reductase [30]. Hereafter, mevalonate is phosphorylated to isopentenyl 5-diphosphate (IPP) [31]. This isoprenoid is the basis for all subsequent isoprenoids (geranyl diphosphate (GPP) and farnesyl diphosphate (FPP)) [30,32]. After another series of sequential enzymatic steps, cholesterol is the main end product of this pathway [30,32]. As a consequence of the large number of enzymatic conversions, a reduction in the mevalonate pathway does not only lower cholesterol biosynthesis, but also affects many other cellular pathways. The reduced production of IPP, for example, leads to lower levels of important molecules for electron transport in mitochondria (e.g., ubiquinone and heme A) [33]. Moreover, decreased protein prenylation due to low FPP and GPPP levels results in decreased activity of central cellular signaling pathways, including Rho and Rac proteins. This has been associated with the beneficial and adverse effects of statins on cardiovascular health, as discussed in more detail below. Similarly, decreased selenoprotein synthesis is observed upon mevalonate pathway inhibition, which produces essential factors for protein selenation. Finally, the cholesterol-lowering activity of statins depends on several effects independent of the mevalonate pathway, including an increased LDL receptor activity due to reduced cholesterol synthesis in the liver. This, in turn, causes an increased re-uptake of LDL cholesterol from the circulation with subsequent cholesterol lowering as observed in clinical practice [34]. Statins also possess an inhibitory effect on apolipoprotein B-100 synthesis in the liver, thereby reducing the synthesis and secretion of triglyceride-rich lipoproteins into the circulation [35].

## 3. Statins Beneficially Affect the Diseased Heart

**Figure 2:**
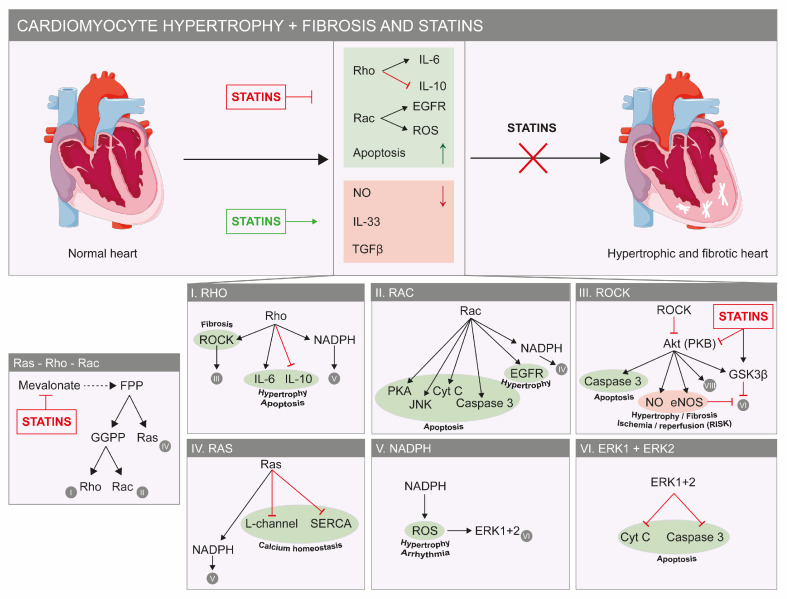
**Beneficial effects of statins on the heart.** Factors that stimulate cardiac hypertrophy and fibrosis are indicated in green and in red for factors that reduce cardiac hypertrophy and fibrosis (**top**). Statins inhibit hypertrophic and fibrotic pathways, as they have a stimulatory effect on factors that reduce both pathologies (red factors). The molecular details of these pathways are shown. In green, factors that stimulate or deteriorate hypertrophy, apoptosis, fibrosis and calcium homeostasis are shown. In red, factors that inhibit hypertrophy or fibrosis (**bottom**) are shown. The panel most to the left shows part of the mevalonate pathway and the way statins inhibit the important Ras, Rho and Rac proteins. Abbreviations: Cyt C: cytochrome *c*; EGFR: epidermal growth factor; FPP: farnesyl pyrophosphate; GGPP: geranylgeranyl-pyrophosphate; eNOS: endothelial nitric oxide synthase; ERK: extracellular signal-regulated protein kinase; GSKβ: glycogen synthase kinase 3 β; JNK: *c*-Jun N-terminal kinase; NADPH: nicotinamide adenine dinucleotide phosphate; NO: nitric oxide; PKA: protein kinase A; PKB: protein kinase B; ROS: reactive oxygen species; TGFβ: transforming growth factor-β; SERCA: sarcoplasmic reticulum Ca^2+^ ATPase.

The pleiotropic effects of statins on the cardiovascular system have recently been extensively reviewed [36]. They emphasize the role of statins in modulating different pathological mechanisms in various cell types of the cardiovascular system, including cardiomyocytes, which are associated with reductions in cardiac hypertrophy and fibrosis [36]. These effects are most likely mediated via inhibition of Rho proteins, decreased apoptosis and increased nitric oxide (NO) availability (Figure 2). As Rho proteins need to be prenylated by GGPP, the reduction in myocardial fibrosis could be predominantly explained by on-target statin-induced inhibition of the mevalonate pathway. This is further supported by the fact that other drugs inhibiting this pathway (e.g., alendronate and fasudil) demonstrated similar, but less prominent beneficial effects in vitro [37]. In myocardial infarction, statins have also proven to reduce infarct size via the mevalonate pathway in rats and mice [36,38]. A central role for the pathophysiological role of this pathway is further supported by increased cardiomyocyte hypertrophy, induction of apoptosis and decreased mitochondrial membrane potential upon overexpression of farnesyltransferase β, a downstream enzyme of the mevalonate pathway in vitro [39]. Besides interfering with Rho proteins and NO signaling, inhibition of the mevalonate pathway by statins also leads to de-differentiation of myofibroblasts in vitro [40]. This reverts the differentiation of fibroblasts to myofibroblasts, which is seen as a key process in cardiac fibrosis. Similar effects have been observed in statin-treated rats with cardiac fibrosis, which also revealed a mechanistic role for cardiomyocyte-derived exosomes in fibroblast to myofibroblast differentiation [41]. Inhibition of Rho leads to a decreased IL-6 secretion, which also contributes to the beneficial effects of statins on cardiac hypertrophy, as IL-6 is associated with reduced left ventricular mass in hypertensive patients (Figure 2) [42,43]. Concomitantly, an increased IL-10 release contributes to improved post-infarction remodeling in vitro and in rats [44,45,46]. Stimulation of other cytokines, including IL-33 and IL-1, is linked to the anti-hypertrophic and anti-apoptotic effects of statins in human cardiomyocytes [47]. Similar anti-inflammatory and anti-apoptotic effects have been linked to inactivation of the TLR-4/NF-κB pathway, which in a concerted action with dendritic cells leads to suppression of inflammation, fibrosis and apoptosis in vitro [48]. Finally, rosuvastatin prevents periprocedural myocardial infarction both in vitro and in vivo through inhibition of the NLRP3 inflammasome, a molecular platform triggering activation of inflammatory caspases [49]. Lately, the role of the anti-inflammatory effects of statins on myocarditis has been extensively reviewed [50]. Primarily studied in vivo, statins reduce the severity of myocarditis through altering T-cell-mediated immune responses [50].

Inhibition of the mevalonate pathway by statins also leads to lower Rac activity, a subfamily of the Rho GTPases (Figure 2). This mechanism has been associated with long-term improvement of the ventricular ejection fraction in heart failure patients, which can be explained by a reduced production of reactive oxygen species (ROS) driven by Rac [51,52]. The etiologic importance of this mechanism is emphasized by the negative correlation between increased ROS and the severity of the left ventricular ejection fraction in humans [51,53,54]. Consistent with this, short-term simvastatin treatment of patients with non-ischemic cardiomyopathy improved their left ventricular ejection fraction by 7 percent [55]. Long-term exposure to atorvastatin in rabbits decreased in vitro vulnerability to oxidative stress induced by ROS exposure [56]. This study also showed positive effects by a decreased oxygen consumption in cardiac muscle fibers, which could potentially be explained by decreased mitochondrial ROS production [56]. Similar effects are also associated with the beneficial effects of rosuvastatin on periprocedural myocardial infarction in mice, both in vitro and in vivo [49]. A decreased oxidative phosphorylation capacity may also underly the protective effect of statins on myocardial ischemia/reperfusion damage, which is associated with a reduction in ATP after reperfusion as was seen in dogs [57]. In ischemic/infarcted areas, statins positively modulate and activate endogenous cardiac stem cells in vitro and in rats and mice, obviating the need for allogenic stem cell transplantation [27,58]. This mechanism contributes to cardiac remodeling after myocardial infarction, and was also observed with chronic statin use in rodents [28,59]. In the ARMYDA study, statins have proven to reduce reperfusion injury after coronary intervention and similar effects were observed in patients undergoing bypass surgery [60,61]. It has also been proven that statins do not influence troponin release under extreme strenuous conditions (marathon running) [62].

Although we mainly focus on cardiomyocytes, it is important to briefly discuss the role of endothelial cells in the pleiotropic effects of statins on cardiac remodeling (i.e., hypertrophic and fibrotic changes). Inhibition of transforming growth factor-β 1 (TGFβ1) or induction of the endothelial Krüppel-like Factor 2 (Klf2)–TGFβ1 pathway with subsequent inhibition of TGFβ1 appears to be essential for the protective effects of statins on fibroblast proliferation and myofibroblast formation in mice [63,64]. Inhibition of the epidermal growth factor receptor (EGFR) signaling pathway by statins reduces blood pressure and pressure overload with slower onset of heart failure and amelioration of cardiac remodeling in vivo [65,66].

Besides on-target effects downstream the mevalonate pathway, statins also exert beneficial effects via various off-target mechanisms affecting myocardial ion homeostasis. Recently, rosuvastatin was demonstrated to modulate the β-adrenergic signaling cascade in rat cardiac myocytes, resulting in reduced L-type calcium currents and lower rates of contraction and relaxation (Figure 2) [67]. Moreover, intracellular free calcium plays an important role in cardiac functioning, and its deregulation causes arrhythmias. As statins lower oxidized low-density lipoprotein (oxLDL) levels, disturbed calcium handling because of high oxLDL is attenuated in humans [68]. The importance of calcium regulation is probably most prominent in atrial fibrillation, as recently reviewed elsewhere [69]. Besides calcium signaling, intracellular potassium levels are also beneficially affected by statins. Most attention has been paid to the effects on ATP-sensitive potassium (K_ATP_) channels, which are necessary for cellular protection during metabolic stress. Decreased expression of K_ATP_ channels in the presence of cholesterol is most likely regulated via the sterol response element-binding protein (SCREBP) family and thereby directly affected through the cholesterol-lowering action of statins in vitro [70]. Moreover, K_ATP_ channels play a pivotal role in the pro-survival pathway RISK (reperfusion injury salvage kinase) by closure of the mitochondrial permeability transition pore (MPTP) [71]. Closing of MPTP is a way of protecting the myocardium against ischemia/reperfusion damage and is established by increased levels of extracellular signal-regulated protein kinase (ERK) and Akt, which are both activated by statins (Figure 2) [71,72].

To conclude, statins hold great promise for their beneficial effects in various cardiovascular pathologies in addition to their cholesterol-lowering effects. However, it is important to note that the results in humans were all obtained in small or non-randomized retrospective studies and should therefore be interpreted with care [73]. Two larger randomized, double-blind, placebo-controlled trials (GISSI-HF and CORONA) did not find any therapeutic effects of statins on heart failure endpoints [2,74]. Interestingly, both trials studied rosuvastatin, a known hydrophilic statin with fewer possible pleiotropic effects. Moreover, a low starting dose of 10 mg was used, whereas most studies found positive effects with higher dosing regimens [75]. Finally, the statin-induced anti-inflammatory response might play a less prominent role in the pathophysiological mechanism of heart failure [76]. This warrants further clinical validation of the supposed molecular mechanisms described above.

## 4. Undesired Effects of Statins on Cardiomyocytes

In contrast to the vast number of beneficial effects of statins on the heart, it is important to understand the mechanisms behind the possibly undesired, subclinical effects to explain the salient difference with skeletal muscle side effects.

**Figure 3:**
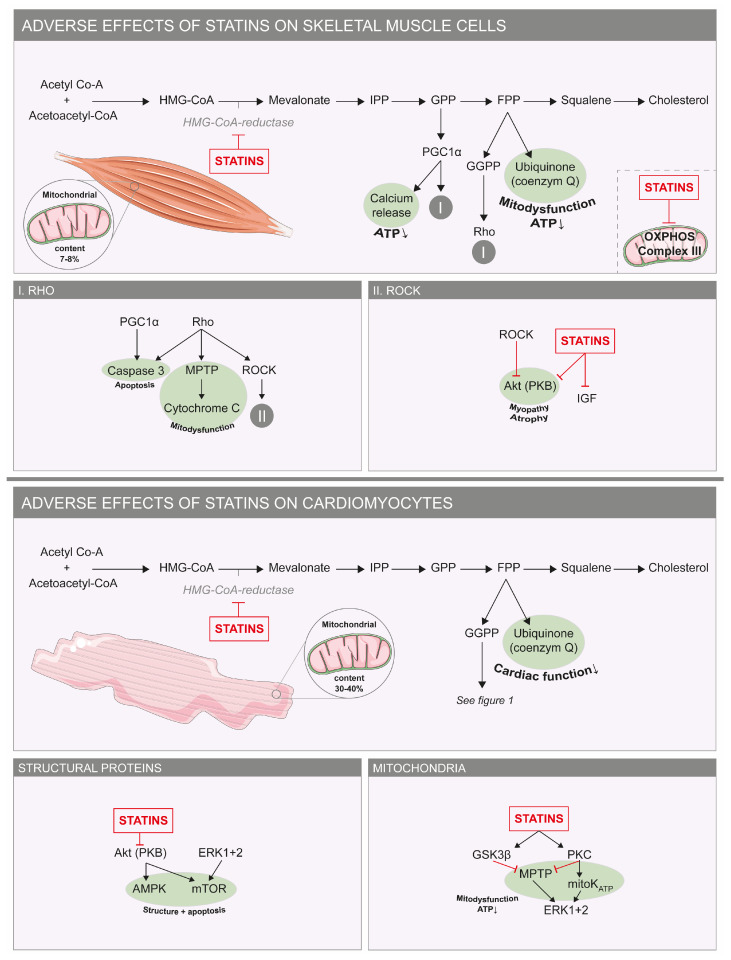
**Adverse effects of statins on skeletal and cardiac muscle cells.** Molecular mechanisms underlying the adverse effects of statins on skeletal muscle cells (**top**) and cardiomyocytes (**bottom**). In green are factors that stimulate apoptosis and mitochondrial dysfunction. On the right are small boxes with details of the pathways seen in the larger boxes. Abbreviations: Akt: PKB/protein kinase B; AMPK: adenosine monophosphate-activated protein kinase; ATP: adenosine triphosphate; ERK: extracellular signal-regulated protein kinase; FPP: farnesyl pyrophosphate; GGPP: geranylgeranyl-pyrophosphate; GPP: geranyl pyrophosphate; GSK3β: glycogen synthase kinase 3 β; IPP: isopentenyl pyrophosphate; MPTP: mitochondrial permeability transition pore; mTOR: mechanistic target of rapamycin; OXPHOS: oxidative phosphorylation; PGC1α: peroxisome proliferator-activated receptor gamma coactivator 1-α; PKC: protein kinase C.

Recently, an adverse influence of atorvastatin, but not pravastatin, on cardiomyocyte function has been described [24]. In cardiomyocytes, but also upon long-term (i.e., 7 weeks) in vivo exposure in mice, inhibition of Akt/mTOR signaling (Figure 3) alters cardiac and mitochondrial ultrastructure. mTor is a protein vital for the regulation of cellular processes including preservation of cardiovascular integrity and function under stressful conditions and adaption to mechanical, ischemic and age-induced cardiac injury [77]. This observation seems to contradict the reported Akt/mTOR activation upon 1 week administration of rosuvastatin in mice, as described above, but this could be explained by the differential regulation of mTOR via mitochondrial ROS production [78]. Low ROS levels are known to activate this pathway [78], whereas high to moderate ROS levels may be the result of long-term exposure to atorvastatin, leading to alterations in cardiac mitochondrial ultrastructure and mTOR inhibition [24]. The difference in inhibitory potency against mTOR between rosuvastatin and pravastatin may be related to their different ability to enter cells (due to different compound lipophilicity and HMGR inhibitory potency) [79], as we have also observed for the absence of an inhibitory effect of pravastatin on mitochondrial activity in C2C12 myoblasts [19]. Moreover, lipophilic statins like lovastatin and simvastatin significantly increased mortality in cardiomyopathic hamsters and enhanced the stunning of myocardium in ischemic dogs [57,80]. In humans, statins significantly enhanced cardiac troponin release after moderate exercise [81]. However, this was only seen in participants without any diagnosis of coronary artery disease and using highly sensitive troponin measurements.

Mitochondria not only play a pivotal role in statin-induced adverse skeletal muscle effects, but also seem to be involved in adverse effects of statins on human cardiac function, as has been shown by reduced plasma CoQ_10_ levels (Figure 3) [24,82,83]. Furthermore, CoQ_10_ supplementation could reverse statin-induced worsening of diastolic cardiac function and was shown to be effective in the treatment of statin-induced cardiomyopathy [84,85]. The suggestion that the life-saving statins could induce heart failure or cardiomyopathy is intriguing but also controversial as a significant clinical substrate lacks. Although the prevalence of heart failure, especially with preserved ejection fraction as is seen with diastolic dysfunction, has risen within the past decades of increased statin usage, and other factors like increased aging and obesity could also account for this, a contributing role for statins has not been fully eliminated [86]. It would therefore be worth considering to explore this hypothesis further in a randomized manner [87].

Next to a negative effect of mitochondrial dysfunction on cellular redox status, the decreased production of selenoproteins, which depends on the mevalonate pathway for their biosynthesis, could further impair this status. The selenoproteome is known to be vital for the anti-oxidative capacity of the cell [82,88]. Interestingly, selenium deficiency (Keshan disease) is related to the development of heart failure, which supports the idea that decreased selenoprotein synthesis is a mechanism that could underly statin-induced cardiomyocyte dysfunction.

Although statins do not seem to have a clinically relevant negative effect on the heart, co-administering statins with other drugs that impede mitochondrial function could shift this towards a clinical manifestation, as it may result in an additive effect on cardiac function. For example, atorvastatin use potentiated the adverse cardiac effects of the β-adrenergic agonist isoproterenol, which is characterized by augmented endothelial dysfunction, induction of oxidative stress and enhanced inflammatory and apoptotic pathways in rats [89]. Second, elderly people may be more at risk for the mitochondrial toxicity of drugs, including statins, as mitochondrial content and function decline with age [90]. Finally, underlying cardiac morbidity could affect mitochondrial function and put these patients at risk for adverse effects of statins. A recent critical appraisal on the possible adverse effects of statins on the heart discussed the idea that statins may be toxic to mitochondria leading to impaired ATP production and heart failure [82,91]. The authors suggested that rather than the dosage only, the prosarcopenic (i.e., stimulation of muscle wasting) properties of statins also determine whether they have favorable or detrimental effects in failing hearts, as these mechanisms show a high molecular similarity with sarcopenia in heart failure [91].

## 5. Mitohormesis 2.0: Explaining the Difference between Statin Adverse Effects on Skeletal and Cardiac Myocytes

Although statins have shown some negative effects on cardiac muscle tissue in vitro, no clinically relevant effects have been found in animal models and in small human cohort studies [24,81,92]. Here, we discuss previous theories proposed to explain these differences (i.e., so-called “mitohormesis” hypothesis) [26] and append it with novel alternative explanations based on pharmacokinetic, morphologic, physiological and metabolic differences between skeletal and heart muscle (i.e., “mitohormesis 2.0” hypothesis) [26].

**Figure 4:**
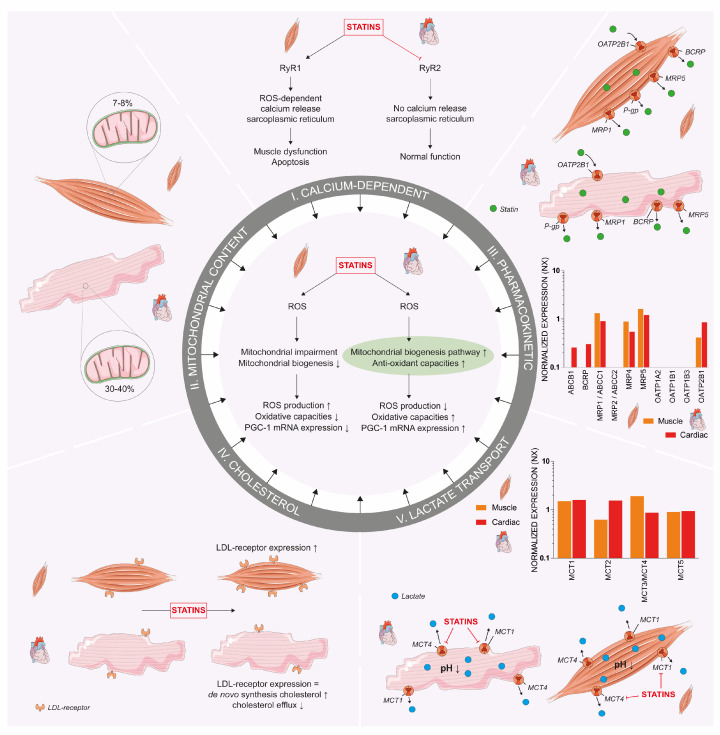
**Mitohormesis 2.0.** The proposed mechanisms behind the metabolic differences between cardiac and skeletal muscle cells. Central to the figure is the previously described mitohormesis theory. Besides this difference, other proposed mechanisms include differences in calcium-dependent ROS, mitochondrial content, transporter expression resulting in different rates of influx and efflux of statins, cholesterol homeostasis and MCT expression. Together, these variations in mechanisms are proposed as a new mitohormesis (i.e., mitohormesis 2.0) theory. Numbers on mRNA protein expression were obtained from the Human Protein Atlas and displayed on a logarithmic scale. Abbreviations: BCRP: breast cancer resistance protein; LDL: low-density lipoprotein; MCT: monocarboxylate transporter; MRP: multidrug resistance-associated protein; NO: nitric oxide; OATP: organic-anion-transporting polypeptides; PGC-1: peroxisome proliferator-activated receptor gamma coactivator 1-α; P-gp: P-glycoprotein; ROS: reactive oxygen species; RyR: ryanodine receptor. An increase is displayed as upward arrow and a decrease as downward arrow.

First, atorvastatin has been shown to activate cardiac mitochondrial biogenesis through ROS production, which leads to an increase in anti-oxidative capacity. However, the opposite is seen in skeletal muscles where ROS production negatively affects mitochondrial biogenesis and capacity [26]. This “mitohormesis” theory proposed by Bouitbir and colleagues, where a non-lethal stressor (statin) induces a response in mitochondria (low concentration of ROS resulting in PGC-1 expression) to increase stress resistance, is centered around the differences in ability to cope with oxidative stress and PGC-1α-dependent mitochondrial biogenesis (Figure 4) [26].

Second, statin-induced ROS production has been shown to provoke calcium release from the sarcoplasmic reticulum through the dissociation of the stabilizing protein FK506-binding protein (FKBP12) of the calcium release channel (ryanodine receptor (RyR) 1) [93]. This results in a calcium leak, skeletal muscle dysfunction and elevated pro-apoptotic signaling, which are not observed in cardiomyocytes [93]. This study also demonstrated the association of this mechanism, as well as the induction of mitochondrial biogenesis, with mitigation of the deleterious effects of statins on skeletal muscle cells upon moderate exercise. The difference in ROS-dependent Ca^2+^ release was associated with a different interaction of statins with RyR, as they activated RyR1 in muscles in the open status with increased calcium spark frequency, whilst inhibiting the RyR2 in cardiac muscles with lower calcium sparks in vitro (Figure 4) [94]. Although the structures of RyR1 and RyR2 are similar for approximately 65%, the small differences relate to special functions of each isoform [95,96]. Moreover, both the acid and lactone form of statins act upon RyR1, suggesting that the difference between the statin-induced activation of RyR1 and inhibition of RyR2 does not depend on their pharmacophore [94].

Third, we propose that differences in baseline mitochondrial content between cardiomyocytes and skeletal muscles could explain the capacity of both tissues to cope with statin-induced mitochondrial dysfunction [19,24]. A mitochondrial content in the range of 7–8% in skeletal muscle tissue is far lower compared to cardiomyocytes in which 30–40% of the cells consist of mitochondria (Figure 4) [97]. This also translates into a higher maximal respiratory capacity in cardiac tissue, which is thought to not be different from skeletal muscle when corrected for mitochondrial mass [98]. The difference in mitochondrial oxidative capacity suggests a larger reserve capacity in cardiomyocytes. As a result, it can be expected that cardiac myocytes have an enhanced potential to cope with statin-induced mitochondrial effects.

Fourth, we hypothesize that differences in the cellular uptake and efflux of statins in muscle and cardiac tissue could play a role. Cellular uptake of statins is mediated by various members of the solute carrier organic anion transporter (OATP) family, including OATP1A2, OATP1B1, OATP1B3 and OATP2B1 [99]. Although statin transport by OATP1B1 into hepatocytes has been most intensively studied, OATP2B1 is the only transporter expressed in skeletal muscle tissue and as such is expected to be relevant for statin-induced muscle complaints [99]. Remarkably, OATP2B1 gene expression is 2.7 times higher in cardiac compared to skeletal muscle tissue (based on normalized transcriptomic expression scores, Human Protein Atlas) (Figure 4) [100]. OATP transporters seem to be essential for the import of statin acid forms, but not for the lactone metabolites, which can freely diffuse across the plasma membrane. Statin lactones have been shown to be more myotoxic than their acid forms by us and others [19,101]. Statin lactones can be actively transported out of the cell by P-glycoprotein (PgP) and breast cancer resistance protein (BCRP) [102,103], whereas statin acid forms are removed by multidrug resistance-associated protein (MRP) 1 and MRP2 [99]. Both PgP and BCRP expression is higher in cardiac compared to skeletal muscle tissue [100], which could provide an explanation for the lower susceptibility of cardiomyocytes to adverse statin effects, because of the higher capacity to remove the toxic lactone metabolites.

Fifth, there is a pronounced difference in cellular cholesterol homeostasis between cardiomyocytes and skeletal muscle (Figure 4). Cardiomyocytes primarily use de novo-synthesized cholesterol or cholesteryl–ester pools for cellular maintenance, as they poorly internalize LDL [104,105,106]. The increased reliance on systemic cholesterol by skeletal muscle cells is emphasized by the observation that in vivo statin treatment increased LDL receptor expression and cholesterol uptake in skeletal muscles, but not in cardiac muscle [106]. In line with this idea, cardiomyocytes use their cholesteryl ester pool to keep a constant level of free cholesterol without upregulating LDL-cholesterol uptake receptors upon statin treatment [105,106]. In addition, efflux of cholesterol is lower in cardiomyocytes without a downregulation of the efflux transporters involved (i.e., ABCA1 and ABCG1) [105], which could be explained by a reduced functionality of these transporters through inhibition by statins [105,107,108]. Consequently, cardiomyocytes may be less prone to the harmful effects of low plasma cholesterol levels compared to skeletal muscle.

Sixth, the inhibition of monocarboxylate transporter (MCT) could also play a role, as statins have shown to inhibit MCT1 and 4, which associates with statin-induced skeletal muscle complaints through intracellular accumulation of lactate, leading to adverse intracellular acidification [109]. Although MCT1 is ubiquitously expressed, MCT4 expression is 11-fold lower in cardiac tissue compared to skeletal muscle (Figure 4). Therefore, statin-induced MCT inhibition is less prominent in cardiac tissue. Though, it remains unknown whether statins also inhibit other MCT isoforms, including MCT2, which has an 8-fold higher expression in cardiomyocytes. Further research is warranted to elucidate whether statins could negatively affect cardiac cellular metabolism by MCT inhibition, or whether differential MCT4 expression indeed determines the difference between statin effects in skeletal and cardiac muscle.

## 6. Outlook

Statins have proven to be very effective cholesterol-lowering drugs, which has contributed to a substantial reduction in cardiovascular morbidity and mortality. Their beneficial effects have been further extended by an increasing number of pleiotropic cardiovascular effects that have been proposed over the past decades. Through the inhibition of Rho, NO and ROS, statins have, for example, been shown to reduce hypertrophy, fibrosis, apoptosis and infarct size in the diseased heart. However, most of these results have been obtained in in vitro and animal studies, and human trials have so far been underpowered due to a limited number of patients or single-center studies [110,111,112]. Therefore, larger randomized clinical trials are needed to fully validate this therapeutic potential of statins as treatment for patients with hypertrophic cardiomyopathy or reducing infarct size in the acute setting.

Although a decreased metabolic capacity after long-term statin treatment seems to play a role in skeletal muscle complaints, the clinical phenotype in the heart is different. The differences between skeletal muscle cells and cardiomyocytes remain interesting and could hence provide leads to obtain better insight into the pathomechanisms of the potential adverse effects in both cell types. Here, we have discussed five possible mechanisms that could explain the difference in statin effects on cardiac and skeletal muscle, including, variations in the regulation of mitochondrial biogenesis and redox balance, difference in baseline mitochondrial content, variability in membrane transporters mediating cellular statin uptake and efflux, differences in cholesterol homeostasis and variability in lactate transport (i.e., mitohormesis 2.0 hypothesis).

Understanding of the mitohormesis 2.0 theory may help to clarify the clinical relevance of the potential beneficial and adverse effects of statins on the heart. Specifically, focusing on human cellular models, including induced pluripotent stem cell (iPSC)-derived cardiomyocytes, could be helpful.

Since the potential harmful cardiac effects of statins emerging from several in vitro and in vivo animal studies are not observed clinically, there is a need to better understand the underlying mechanisms, especially those that protect the heart. Further research should also be directed to vulnerable groups, including the elderly and patients with heart disease, who may be more susceptible to the adverse effects of statins because of impaired mitochondrial function. These insights may help in the development of safer cardiovascular drugs and a more personalized statin treatment.

## Data Availability

No new data were created or analyzed in this study. Data sharing is not applicable to this article.
